# Supplementary material for: Comparative genomics provides new insights into the diversity, physiology, and sexuality of the only industrially exploited tremellomycete: Phaffia rhodozyma
Source: BMC Genomics. 2016 Nov 9;17:901. doi: 10.1186/s12864-016-3244-7 (PMC5103461; doi:10.1186/s12864-016-3244-7)
Supplement: Additional file 6: — List of orphan genes with links to PFAM (related to Additional file 1: Table S1). (ZIP 1428 kb) [file 12864_2016_3244_MOESM6_ESM.zip › BLAST_HTML_FTR/G01744_P.html]

BLAST Search Results


```
BLASTP 2.2.27+


Reference:
Stephen F. Altschul, Thomas L. Madden, Alejandro A. Schäffer,
Jinghui Zhang, Zheng Zhang, Webb Miller, and David J. Lipman (1997),
"Gapped BLAST and PSI-BLAST: a new generation of protein database
search programs", Nucleic Acids Res. 25:3389-3402.


Reference for
composition-based statistics:
Alejandro A. Schäffer, L. Aravind, Thomas L. Madden, Sergei
Shavirin, John L. Spouge, Yuri I. Wolf, Eugene V. Koonin, and
Stephen F. Altschul (2001), "Improving the accuracy of PSI-BLAST
protein database searches with composition-based statistics and
other refinements", Nucleic Acids Res. 29:2994-3005.


Database: nr
           71,551,133 sequences; 26,053,659,533 total letters


Query= G01744_P

Length=332
                                                                      Score     E
Sequences producing significant alignments:                          (Bits)  Value

emb|CDZ96188.1|  hypothetical protein [Xanthophyllomyces dendrorh...   657    0.0  
gb|KIR50874.1|  septation protein imp2 [Cryptococcus gattii Ru294]    42.4    0.74 
gb|KIY31244.1|  septation protein imp2 [Cryptococcus gattii E566]     39.7    5.1  
gb|KIR76855.1|  septation protein imp2 [Cryptococcus gattii EJB2]     39.7    5.2  
gb|KJE01410.1|  septation protein imp2 [Cryptococcus gattii NT-10]    39.7    5.4  
ref|XP_003192417.1|  hypothetical protein CGB_B9120W [Cryptococcu...  39.7    5.5  


 >emb|CDZ96188.1| hypothetical protein [Xanthophyllomyces dendrorhous]
Length=331

 Score =  657 bits (1696),  Expect = 0.0, Method: Compositional matrix adjust.
 Identities = 330/331 (99%), Positives = 330/331 (99%), Gaps = 0/331 (0%)

Query  1    MNPTAVCSPTSTPSSPRLHSSHSSPQTTAIAASTTAGGFYMKSPSYPSTPAPHYRTSIHS  60
            MNPTAVCSPTSTPSSPRLHSSHSSPQTTAIAASTTAGGFYMKSPSYPSTPAPHYRTSIHS
Sbjct  1    MNPTAVCSPTSTPSSPRLHSSHSSPQTTAIAASTTAGGFYMKSPSYPSTPAPHYRTSIHS  60

Query  61   SHFPPQTPPPGYTHPPPPPSHSHPSLSNRQQGQRHGHSRSNSNSSIYHSYQYQHNQSHPY  120
            SHFPPQTPPPGYTHPPPPPSHSHPSLSNRQQGQRHGHSRSNSNSSIYHSYQYQHNQSHPY
Sbjct  61   SHFPPQTPPPGYTHPPPPPSHSHPSLSNRQQGQRHGHSRSNSNSSIYHSYQYQHNQSHPY  120

Query  121  PFPSTSMVDPVSPGGLTGLGFDSANPDRQVVHLDRLDVSELSRALRRARAGMGYQQLDGD  180
            PFPSTSMVDPVSPGGLTGLGFDSANPDRQVVHLDRLDVSELSRALRRARA MGYQQLDGD
Sbjct  121  PFPSTSMVDPVSPGGLTGLGFDSANPDRQVVHLDRLDVSELSRALRRARAVMGYQQLDGD  180

Query  181  EEDIEEGVEGEREESLPSYRSRGRGVYGSSRSTLLTSFSTGLPHPLPSSSFPSDSTYRPN  240
            EEDIEEGVEGEREESLPSYRSRGRGVYGSSRSTLLTSFSTGLPHPLPSSSFPSDSTYRPN
Sbjct  181  EEDIEEGVEGEREESLPSYRSRGRGVYGSSRSTLLTSFSTGLPHPLPSSSFPSDSTYRPN  240

Query  241  PNHISSTSFQSGPSRRVSCLPSAQTGPFFTEGTSPILCPLEDAHRSGEIDLGDGDRDAEE  300
            PNHISSTSFQSGPSRRVSCLPSAQTGPFFTEGTSPILCPLEDAHRSGEIDLGDGDRDAEE
Sbjct  241  PNHISSTSFQSGPSRRVSCLPSAQTGPFFTEGTSPILCPLEDAHRSGEIDLGDGDRDAEE  300

Query  301  DDVRVSGSWVIWGLGVGLGIGSIGWAYVSHG  331
            DDVRVSGSWVIWGLGVGLGIGSIGWAYVSHG
Sbjct  301  DDVRVSGSWVIWGLGVGLGIGSIGWAYVSHG  331


>gb|KIR50874.1| septation protein imp2 [Cryptococcus gattii Ru294]
Length=1063

 Score = 42.4 bits (98),  Expect = 0.74, Method: Compositional matrix adjust.
 Identities = 41/125 (33%), Positives = 57/125 (46%), Gaps = 5/125 (4%)

Query  13   PSSPRLHSSHSSPQTTAIAASTTAGGFYMKSPSYPSTPAPHYRTSIHSSHFP-PQTPPPG  71
            P S R ++SH   ++   AA +     Y      P++PAP+ RTS   S  P P++PP  
Sbjct  500  PGSVRRNASHRRAESVVSAAGSVRSSMYGHGIKSPASPAPN-RTSFQQSTPPQPRSPPID  558

Query  72   YTHPPPPPSHSHPSLSNRQQGQRHGHSR---SNSNSSIYHSYQYQHNQSHPYPFPSTSMV  128
             T  PPP  H+  +L+      RH  SR   S   S  Y ++      SHP   P+T   
Sbjct  559  STLSPPPGGHTAAALAKSMDEFRHQSSRGPESKRQSVNYSNFADDVVGSHPTSRPTTPSF  618

Query  129  DPVSP  133
             P SP
Sbjct  619  RPASP  623


>gb|KIY31244.1| septation protein imp2 [Cryptococcus gattii E566]
Length=1062

 Score = 39.7 bits (91),  Expect = 5.1, Method: Compositional matrix adjust.
 Identities = 40/125 (32%), Positives = 56/125 (45%), Gaps = 5/125 (4%)

Query  13   PSSPRLHSSHSSPQTTAIAASTTAGGFYMKSPSYPSTPAPHYRTSIHSSHFP-PQTPPPG  71
            P S R ++SH   ++   AA +     Y      P++PAP+ RTS   S  P P++PP  
Sbjct  499  PGSVRRNASHRRAESVVSAAGSMRSSMYGHGIKSPASPAPN-RTSFQQSTPPQPRSPPID  557

Query  72   YTHPPPPPSHSHPSLSNRQQGQRHGHSR---SNSNSSIYHSYQYQHNQSHPYPFPSTSMV  128
             T  PPP  H+  +L+      RH  SR   S   S  Y ++      S P   P+T   
Sbjct  558  STLSPPPGGHTAAALAKSMDEFRHQSSRGPESKRQSVNYSNFADDVVGSQPTSRPTTPSF  617

Query  129  DPVSP  133
             P SP
Sbjct  618  RPASP  622


>gb|KIR76855.1| septation protein imp2 [Cryptococcus gattii EJB2]
Length=1063

 Score = 39.7 bits (91),  Expect = 5.2, Method: Compositional matrix adjust.
 Identities = 40/125 (32%), Positives = 56/125 (45%), Gaps = 5/125 (4%)

Query  13   PSSPRLHSSHSSPQTTAIAASTTAGGFYMKSPSYPSTPAPHYRTSIHSSHFP-PQTPPPG  71
            P S R ++SH   ++   AA +     Y      P++PAP+ RTS   S  P P++PP  
Sbjct  500  PGSVRRNASHRRAESVVSAAGSMRSSMYGHGIKSPASPAPN-RTSFQQSTPPQPRSPPID  558

Query  72   YTHPPPPPSHSHPSLSNRQQGQRHGHSR---SNSNSSIYHSYQYQHNQSHPYPFPSTSMV  128
             T  PPP  H+  +L+      RH  SR   S   S  Y ++      S P   P+T   
Sbjct  559  STLSPPPGGHTAAALAKSMDEFRHQSSRGPESKRQSVNYSNFADDVVGSQPTSRPTTPSF  618

Query  129  DPVSP  133
             P SP
Sbjct  619  RPASP  623


>gb|KJE01410.1| septation protein imp2 [Cryptococcus gattii NT-10]
Length=1063

 Score = 39.7 bits (91),  Expect = 5.4, Method: Compositional matrix adjust.
 Identities = 40/125 (32%), Positives = 56/125 (45%), Gaps = 5/125 (4%)

Query  13   PSSPRLHSSHSSPQTTAIAASTTAGGFYMKSPSYPSTPAPHYRTSIHSSHFP-PQTPPPG  71
            P S R ++SH   ++   AA +     Y      P++PAP+ RTS   S  P P++PP  
Sbjct  500  PGSVRRNASHRRAESVVSAAGSMRSSMYGHGIKSPASPAPN-RTSFQQSTPPQPRSPPID  558

Query  72   YTHPPPPPSHSHPSLSNRQQGQRHGHSR---SNSNSSIYHSYQYQHNQSHPYPFPSTSMV  128
             T  PPP  H+  +L+      RH  SR   S   S  Y ++      S P   P+T   
Sbjct  559  STLSPPPGGHTAAALAKSMDEFRHQSSRGPESKRQSVNYSNFADDVVGSQPTSRPTTPSF  618

Query  129  DPVSP  133
             P SP
Sbjct  619  RPASP  623


>ref|XP_003192417.1| hypothetical protein CGB_B9120W [Cryptococcus gattii WM276]
 gb|ADV20630.1| Conserved hypothetical protein [Cryptococcus gattii WM276]
Length=1063

 Score = 39.7 bits (91),  Expect = 5.5, Method: Compositional matrix adjust.
 Identities = 40/125 (32%), Positives = 56/125 (45%), Gaps = 5/125 (4%)

Query  13   PSSPRLHSSHSSPQTTAIAASTTAGGFYMKSPSYPSTPAPHYRTSIHSSHFP-PQTPPPG  71
            P S R ++SH   ++   AA +     Y      P++PAP+ RTS   S  P P++PP  
Sbjct  500  PGSVRRNASHRRAESVVSAAGSMRSSMYGHGIKSPASPAPN-RTSFQQSTPPQPRSPPID  558

Query  72   YTHPPPPPSHSHPSLSNRQQGQRHGHSR---SNSNSSIYHSYQYQHNQSHPYPFPSTSMV  128
             T  PPP  H+  +L+      RH  SR   S   S  Y ++      S P   P+T   
Sbjct  559  STLSPPPGGHTAAALAKSMDEFRHQSSRGPESKRQSVNYSNFADDVVGSQPTSRPTTPSF  618

Query  129  DPVSP  133
             P SP
Sbjct  619  RPASP  623


Lambda      K        H        a         alpha
   0.313    0.131    0.405    0.792     4.96 

Gapped
Lambda      K        H        a         alpha    sigma
   0.267   0.0410    0.140     1.90     42.6     43.6 

Effective search space used: 2760148359450


  Database: nr
    Posted date:  Sep 23, 2015 12:05 AM
  Number of letters in database: 26,053,659,533
  Number of sequences in database:  71,551,133


Matrix: BLOSUM62
Gap Penalties: Existence: 11, Extension: 1
Neighboring words threshold: 11
Window for multiple hits: 40
```
